# Mortality rates are higher in lewy body and parkinson's disease dementia compared to Alzheimer's dementia in patients referred into a secondary care mental health service. Why?

**DOI:** 10.1192/bjo.2021.698

**Published:** 2021-06-18

**Authors:** Anne K, John O'Brien, Annabel Price, Rudolph Cardinal, Sinead Moylett

**Affiliations:** 1 University of Cambridge, Cambridgeshire and Peterborough NHS foundation trust; 2 University of Cambridge

## Abstract

**Aims:**

We compared survival in four cohorts of dementia patients– Lewy body (LBD), Parkinson's (PDD), Vascular (VD) and Alzheimer's dementia (AD) - in patients referred into Cambridge and Peterborough NHS Foundation Trust (CPFT) mental health services.

Additionally, we investigated reasons for variation in survival in the four cohorts.

**Method:**

Using electronic records we identified retrospective cohorts of patients referred into services from 2013 onwards. Cases of LBD and PDD were identified using text searches, and comparison cohorts with AD or VD identified using ICD10 diagnosis codes ((F00.*) or (F01.*) respectively).

We collected referral (date of referral and service referred into), demographic (date of birth and gender) and diagnosis data on the patients in the four cohorts. Dates of death were available, through central NHS reporting to Trusts.

We used date of first referral as start of the follow-up and end of follow-up, death or 31/12/19. We used Kaplan-Meier and Cox survival analysis to compare survival in the four cohorts.

The cohorts were crossed with Hospital Episode Statistics (HES) data to extract hospital admission diagnoses. We extracted diagnoses of pneumonia due to aspiration and recurrent falls from hospital admissions data using ICD codes (J69.0 and R29.6 respectively). We calculated prevalence of these diagnoses in the dementia groups, in males and females separately.

**Result:**

In Cox analysis (controlling for age at referral, gender and service referred into), the hazard ratio (HR) for death was highest for the PDD group (HR 2.0 (95% CI 1.7–2.4)), followed by LBD (HR 1.4 (95% CI 1.3–1.6)), then VD (HR 1.2 (95% CI 1.0–1.3)), with the AD group as reference. In the same analysis repeated separately for males and females, the highest HR was found in males with PDD (HR 2.3 (95% CI 1.8–2.8)).

Referrals to liaison psychiatry were associated with reduced survival compared to other mental health services (HR 1.7 (95% CI 1.5–2.0)).

The AD cohort showed the lowest rates of pneumonia due to aspiration and recurrent falls in males and in females. The highest rate of pneumonia due to aspiration was found in the male PDD group (27%).

**Conclusion:**

In patients with dementia referred into mental health services, those with AD survive longer compared to other dementia groups, with PDD patients at highest risk of death. Physical frailty including risk of aspiration, is likely to account for some of this difference in survival.

